# Effect of micro-osteoperforation on the rate of canine retraction: a split-mouth randomized controlled trial

**DOI:** 10.1186/s40510-019-0274-0

**Published:** 2019-06-03

**Authors:** Amira A. Aboalnaga, Mona M. Salah Fayed, Noha A. El-Ashmawi, Sanaa A. Soliman

**Affiliations:** Department of Orthodontics, Faculty of Dentistry, Cairo University, 11 Saraya El-Manial Street, Cairo, Egypt

**Keywords:** Micro-osteoperforation, Accelerated orthodontics, Tooth movement, Canine retraction

## Abstract

**Background:**

Among the recent modalities introduced to accelerate orthodontic tooth movement (OTM) is micro-osteoperforations (MOPs), in other words, bone puncturing. The aim of this split-mouth trial was to investigate the effects of MOPs on the rate of OTM.

**Methods:**

Eighteen patients requiring bilateral first premolar extraction and upper canine retraction with maximum anchorage were enrolled in this study. Immediately before canine retraction, three MOPs were randomly allocated to either the right or left sides. MOPs were performed using a mini-screw (1.8 mm diameter, 8 mm length) distal to the canine. Canine retraction continued for 4 months. Data were collected from monthly digital models, in addition to pre- and post-retraction maxillary CBCT images*.* The *primary outcomes* were the rate of canine retraction per month and the total distance moved by the canines. The *secondary outcomes* were the effect of MOPs on anchorage loss, canine root resorption, and pain.

**Results:**

The mean rate of canine retraction in both sides was 0.99 ± 0.3 mm/month. The total distance moved by the canine cusp tip was greater in the MOP than the control side (mean difference 0.06 ± 0.7 mm), which was statistically insignificant (*P* > 0.05(. The total distances moved by the canine center and apex were significantly greater in the MOP than the control side (mean difference 0.37 ± 0.63 mm (*P* < 0.05) and 0.47 ± 0.56 mm (*P* < 0.01) respectively). Insignificant differences were detected regarding anchorage loss and root resorption between both sides (*P* > 0.05). Mild to moderate pain was experienced following the MOP procedure, which rapidly faded away within 1 week.

**Conclusions:**

Micro-osteoperforations were not able to accelerate the rate of canine retraction; however, it seemed to facilitate root movement.

## Background

Orthodontic treatment is well known to be a prolonged one, that is why a considerable number of patients, particularly adults, avoid orthodontic treatments and would rather accept less superior esthetics offered by aligners or fixed prosthodontics. The average orthodontic treatment duration is 2 years [1]. Not only longer treatment duration poses greater risks of gingival inflammation, root resorption [2], and enamel decalcification [3], but most importantly, it can burnout patients’ cooperation [1], which is another factor that detains orthodontic treatment.

Consequently, accelerated orthodontics has gained much popularity in the recent research work. Many modalities have been proposed to accelerate orthodontic tooth movement (OTM) such as low-level laser therapy, corticotomy, interseptal bone reduction, photobiomodulation, or pulsed electromagnetic fields [4]. Among all modalities introduced, corticotomy has the largest number of research evidence supporting its efficacy in OTM acceleration [4] owing to the regional acceleratory phenomenon (RAP). However, it is a relatively invasive procedure with low patient acceptance [5].

Micro-osteoperforation (MOP) is an acceleratory method which has been lately proposed [6]. Unlike corticotomy, this procedure comprises flapless bone puncturing. This minor surgical procedure was first introduced by Teixeira et al. [6], who hypothesized that limited cortical bone perforations were sufficient to elicit RAP, hence accelerating OTM. Nevertheless, later experimental studies [7–10] have shown conflicting evidence regarding its acceleratory effect.

The first human trial conducted by Alikhani et al. [11] reported that MOPs were able to increase the rate of OTM by 2.3-fold. Lately, Alkebsi et al. [12] demonstrated that MOPs were not effective for accelerating OTM; hence, the effect of MOP on OTM still remains indistinct. Recent systematic reviews [13–15] investigating the effectiveness of minimally invasive surgical methods of acceleration emphasized the limited low-quality evidence supporting these procedures and highlighted the urgent need for high-quality randomized controlled clinical trials. Therefore, the primary aim of the current study was to evaluate the effect of MOPs on the rate of OTM in a canine retraction model. The secondary outcomes were the effect of the MOPs on first molar anchorage loss, canine root resorption, and the pain caused by this procedure. The null hypothesis (H0) of this research was that MOPs were not able to accelerate the rate of canine retraction.

## Methods

The current study was a split-mouth randomized controlled trial with 1:1 allocation ratio. The study procedures were approved by the Research Ethics Committee of the Faculty of Dentistry, Cairo University. Eighteen subjects were selected from the outpatient clinic of the Department of Orthodontics, Faculty of Dentistry, Cairo University, who were acquainted with the study procedures and radiation exposures, then written consents were signed. *Registration:* This trial was registered at (ClinicalTrials.gov) with an identifier number: NCT03450278. The protocol was registered after trial conclusion.

The inclusion criteria applied for the participants were adult females ranging between 16 and 30 years requiring bilateral extraction of the maxillary first premolars and canine retraction with maximum anchorage, full permanent dentition with exception of the third molars, good oral hygiene, and periodontal condition. The exclusion criteria were medically compromised patients, chronic use of medications affecting OTM, smoking, or any radiographic evidence of bone loss.

### Sample size calculation

Based on a previous study [16], the mean change in the active and control groups was 1.53 ± 0.67 mm and 0.78 ± 0.24 mm respectively. The sample size was calculated based on type I error probability (0.01), in which the response within each subject group was normally distributed with standard deviation of 0.503. The power analysis showed that 15 subjects were needed to be able to reject the null hypothesis that the population means of the active and control sides were equal with probability (power) 0.9. Considering dropouts, a sample size of 18 patients was considered appropriate.

### Randomization (random number generation, allocation concealment, implementation)

Computer-generated random numbers were generated using Microsoft Office Excel 2007 sheet by a person who was not involved in the clinical trial (MA). The patients’ right sides were randomly assigned to either the MOP or control groups. The numbers of the subjects were kept in opaque sealed envelopes until the commencement of canine retraction. On the day of MOP procedure, subjects were allowed to choose one of the envelopes to detect their number in the randomization sequence and thus detect which was the MOP side.

### Interventions

All subjects received a straight wire appliance (Roth prescription 0.022″ slot bracket system, Ormco-Mini 2000 brackets) on their upper and lower arches excluding the upper first premolars. After initial leveling and alignment, self-drilling temporary anchorage devices (TADs) (Unitek™ TAD, 1.8 × 8 mm) were placed buccally between upper 2nd premolar and first molar bilaterally. Indirect anchorage was prepared by inserting L-shaped 0.019″ × 0.025″ stainless steel wire in the auxiliary tube of the upper first molar bands (cinched back) and connected to the mini-screws with a flowable composite ball. The patient was then referred for extraction of the upper first premolars.

Three months after extraction, complete healing of the extraction socket was assured [17] and the patient was ready to start the canine retraction phase. A pre-retraction (T0) alginate impression and cone beam computed tomography (CBCT) image (CRANEX® 3D – Soredex) were taken for the upper arch, and a rigid stainless steel retraction arch wire 0.017″ × 0.025″ was inserted.

Immediately before canine retraction, canine root length in the MOP side was calculated from the pre-retraction CBCT image using Invivo 5 software version 5.3 (Anatomage, San Jose, CA, USA). An L-shaped wire guide with its vertical segment equal to two thirds of canine root length [18] was ligated to the canine bracket. A permanent marker divided the vertical wire guide into three equal thirds, such that each segment received one MOP. Mesiodistally, the MOPs were performed midway in the extraction space.

**Fig. 1:**
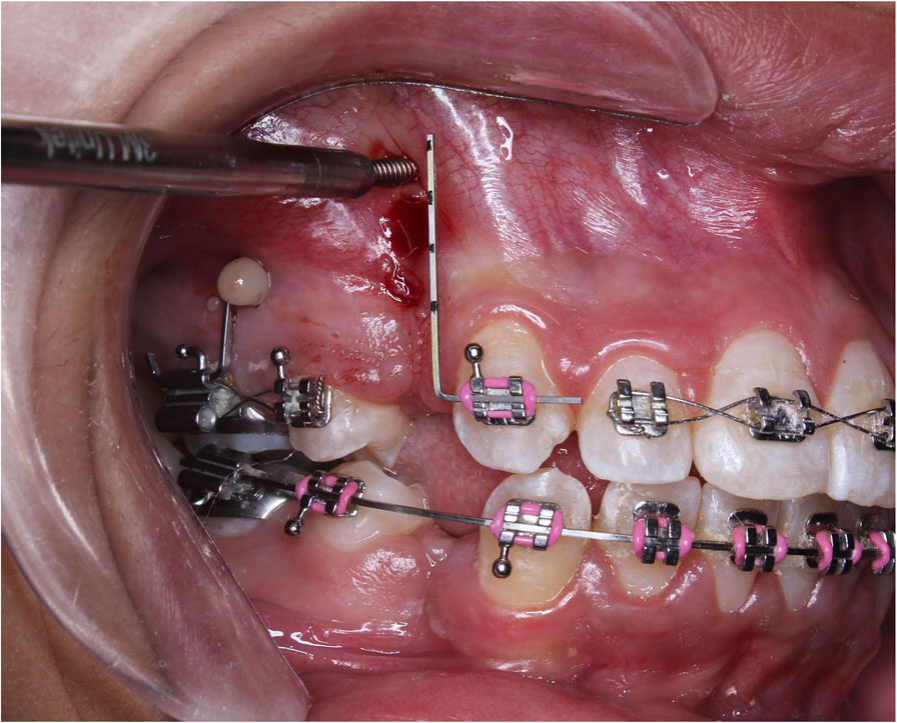
Intra-oral photograph showing the procedure of MOPs using a TAD (note the permanent marks on the vertical segment of the wire guide dividing two thirds of the canine root length into equal thirds, such that one MOP was performed for each third)

After disinfecting the area with betadine, the MOPs were performed under local anesthesia using a TAD (Unitek™ TAD, 1.8 × 8 mm). The TAD was screwed slowly into the alveolar bone, perpendicular to the bone surface, till slight blanching of the surrounding soft tissue was obtained to ensure full-length penetration of the TAD, then the TAD was unscrewed and removed (Fig. 1). Canine retraction was then commenced using NiTi closing coil springs applying 150 g extended between the first molar and canine hooks.

Patients were asked to avoid using painkillers (except acetaminophen if needed) and were given post-operative care instructions. The Numeric Pain Rating Scale, which allows callibrating pain intensity from (1–10) [19], was explained to the patients in order to assess the pain intensity in the postoperative period, immediately after performing the MOPs, 1 day, 3 days, and 1 week after the MOP procedure: 1–3 is considered mild pain, 4–6 is considered moderate pain, and 7–10 is considered severe pain. Patients were also asked to answer the following two questions on the day of the MOP procedure: (1) Which side was more painful at the end of the MOP procedure, the MOP or the control side? (2) Which was more painful, the premolar extraction or the MOP procedure?

Follow-up visits were scheduled every 2 weeks for re-calibrating the springs, checking TADs stability, and detecting any occlusal interferences that may arise during canine retraction. An upper alginate impression was taken every 4 weeks. The study time was continued for 4 months (T1, T2, T3, T4), after which the final upper impression was taken, and the patient was referred to acquire the post-retraction CBCT image.

### Outcomes assessment

**Fig. 2:**
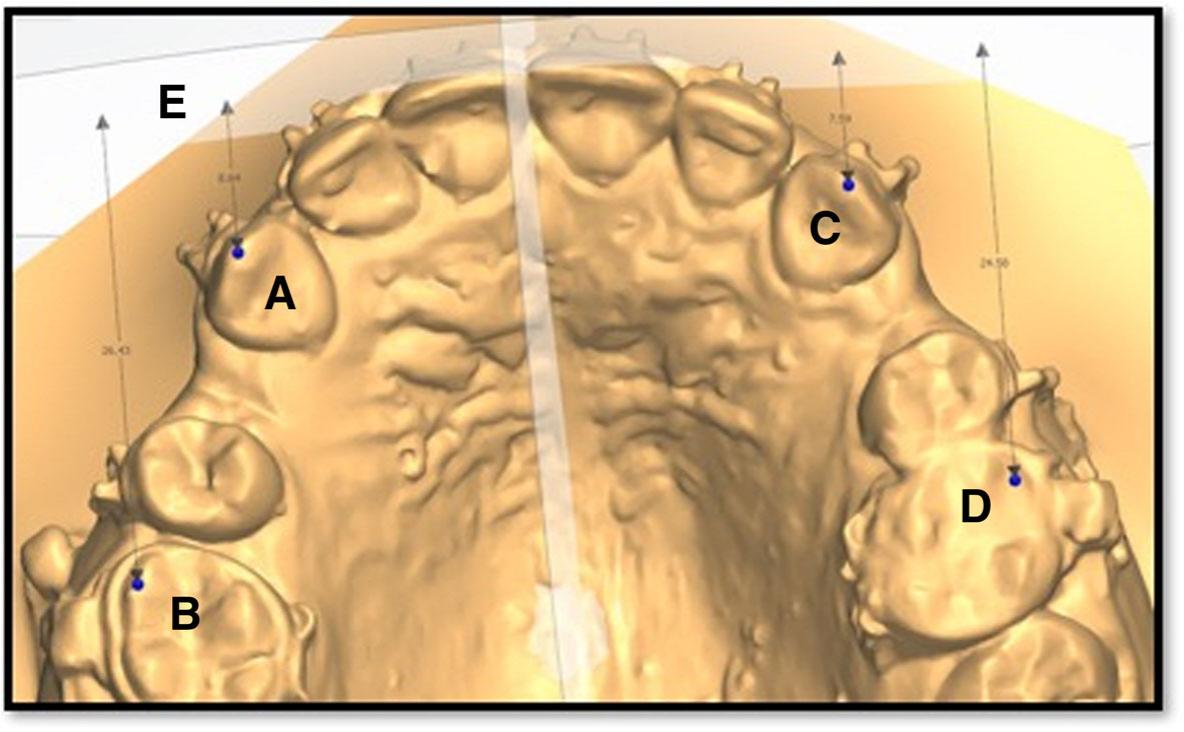
Landmarks used in digital model assessment. **a** Upper right canine cusp tip. **b** MB cusp tip of upper right first molar. **c** Upper left canine cusp tip. **d** MB cusp tip of upper right first molar. **e** Frontal plane

Each upper stone model (T0–T4) was scanned using 3Shape R900 scanner (3Shape A/S, Copenhagen, Denmark) to obtain the .stl format of the digital upper model. Using 3-point superimposition, digital model (T0) was superimposed on the pre-retraction CBCT image using three occlusal points [20] and the frontal plane (FP) was constructed, which was used as a reference plane to detect the rate of canine retraction and anchorage loss. Successive models (T1–T4) were superimposed on model (T0) using three common reference points selected on the third rugae [21]. Using 3 shape software (3-Shape Analyzer), the distance between the upper canine cusp tip and FP in each model (T0–T4) was used to measure the monthly rate of canine retraction, and the sagittal distance between the mesiobuccal (MB) cusp tip of the upper first molar and FP was used to measure anchorage loss (Fig. 2).

**Fig. 3:**
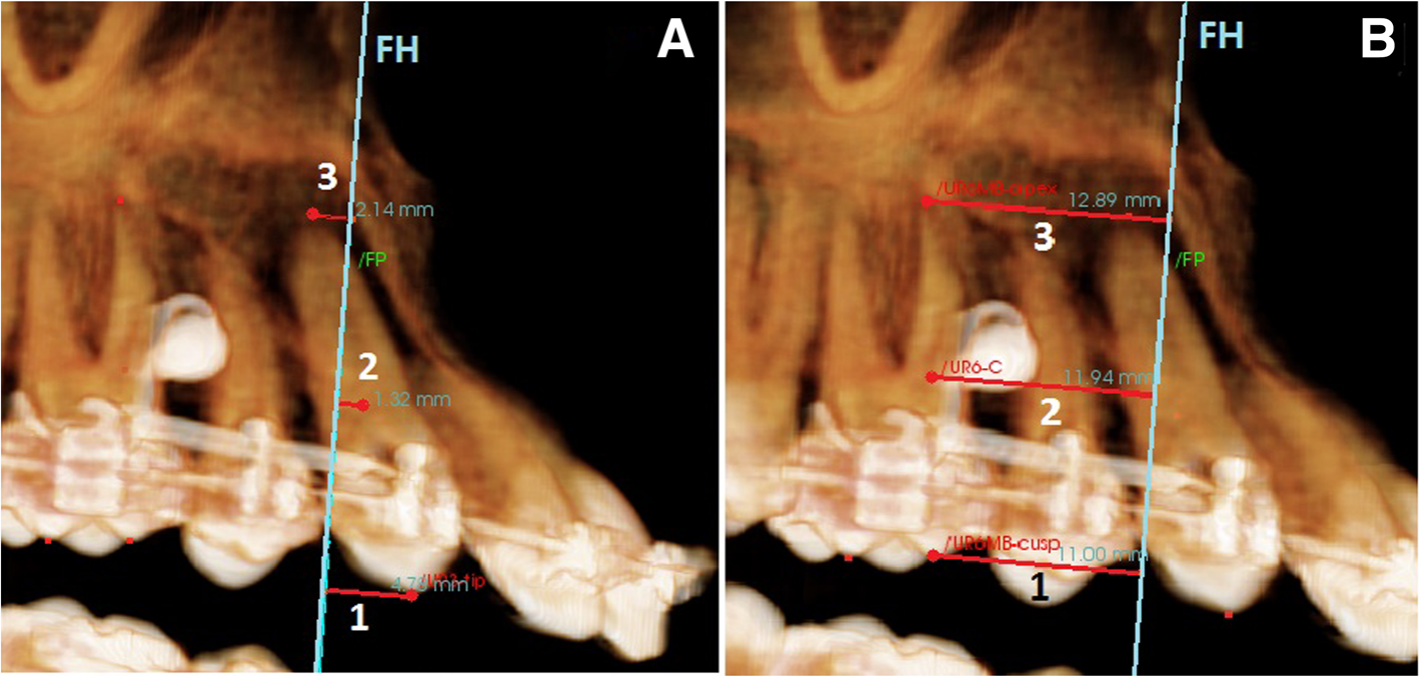
Volumetric CBCT views showing **a** canine retraction measurements; 1: upper right canine cusp tip distance moved (from canine cusp tip to FP), 2: upper right canine center distance moved (from canine center to FP), 3: upper right canine root apex distance moved (from canine root apex to FP). **b** First molar anchorage loss measurements; 1: mesiobuccal (MB) cusp tip loss of anchorage (from MB cusp tip to FP), 2: Center loss of anchorage (from MB root center to FP), 3: MB root apex loss of anchorage (from MB root apex to FP)

Invivo 5 (Anatomage) version 5.3 software was used to calculate the measurements of the pre- and post-retraction CBCT images as shown in (Fig. 3). The total distance moved by the canine and first molar anchorage loss was assessed by comparing the pre- and post-retraction CBCT measurements.

**Fig. 4:**
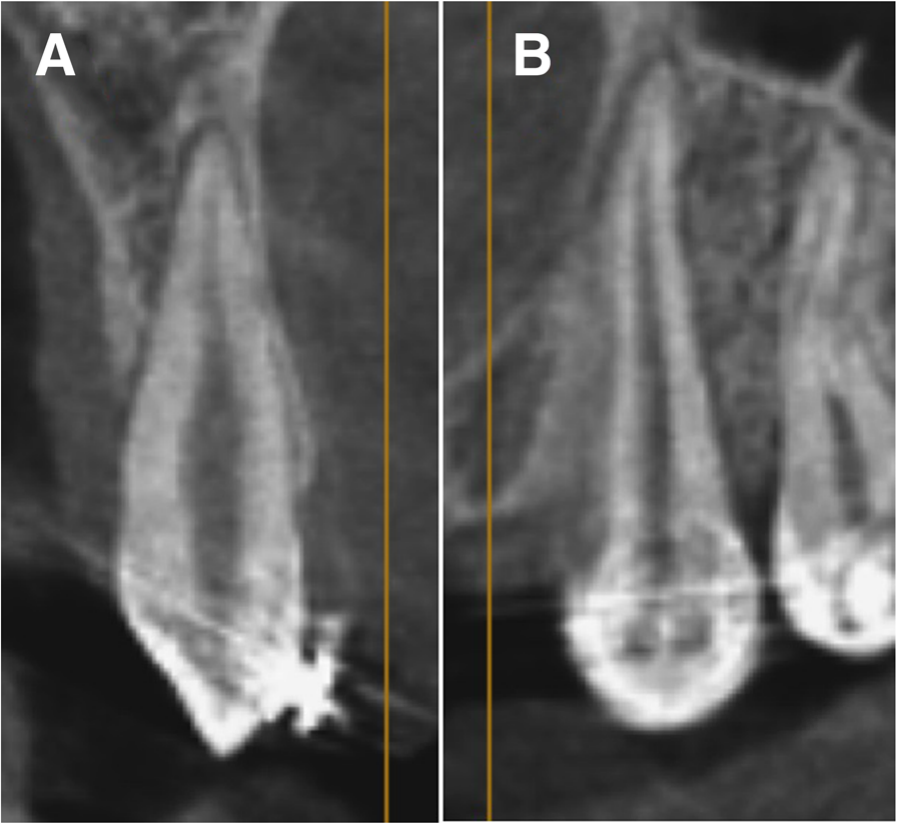
The canine CBCT image reoriented to show the maximum canine root length in **a** the labiolingual cross-section and **b** the mesiodistal cross-section

Root resorption was assessed using the multiplanar view of the Anatomage software. The CBCT image was adjusted using the re-orientation tool, such that the sagittal and the coronal cuts were parallel to the canine long axis. The two cross-sections showing the maximum canine root length were selected for assessment using Malmgren index [22] (Fig. 4)*.* Each canine was given two scores (0–4) according to the root resorption severity detected from the labio-lingual and mesio-distal cross-sections respectively. The difference between the pre- and post-retraction scores for each canine was calculated and evaluated statistically.

The primary assessor (N.A) performed digital models and CBCT measurements blindly for each patient. Ten CBCT images were assessed by the same assessor (N.A) twice, 2 weeks apart, and by another assessor (M.A) once in order to calculate the intra- and inter-observer reliability of the measurements.

### Statistical analysis

Statistical analysis was performed by SPSS (version 17) software package. The variables were described by the mean, standard deviation (SD), range (minimum–maximum), and 95% confidence interval of the mean values. Shapiro-Wilk test of normality was used to determine the appropriate parametric and non-parametric tests. All tested variables were normally distributed; hence, paired sample *t* test was used for comparing the MOP and control sides. Distance moved by canine and assessment of pain changes with time were assessed using one-way repeated measure ANOVA. Chi-squared test of independence was applied for all categorical variables. Significance level was considered at *P* < 0.05 (S). For intra- and inter-observer reliability analysis of all measured variables, Dahlberg Error (DE) and Relative Dahlberg Error (RDE) were used together with concordance correlation coefficients (CCC) including its 95% confidence limits.

## Results

All 18 patients had successfully completed the 4 months duration of the study. The age range of the patients was 16–25 years (mean 20.5 ± 3.85 years). By the end of the fourth month of canine retraction, complete space closure was achieved in 15 patients, 2 patients needed further 1 month of canine retraction, and complete space closure was achieved after 3 months in 1 patient.

All MOP procedures were safely performed without intra-operative surgical complications. Mild gingival inflammation around the TADs was seen in 4 patients (22.2%). Three patients (16.6%) reported mild swelling in the MOP side during the first post-operative week. In 5 patients (27.7%), widening of the mucosal perforation occurred at the most apical MOP, since it was performed in non-keratinized alveolar mucosa. Complete healing occurred after 2 weeks.

### Results of digital model assessment

**Fig. 5:**
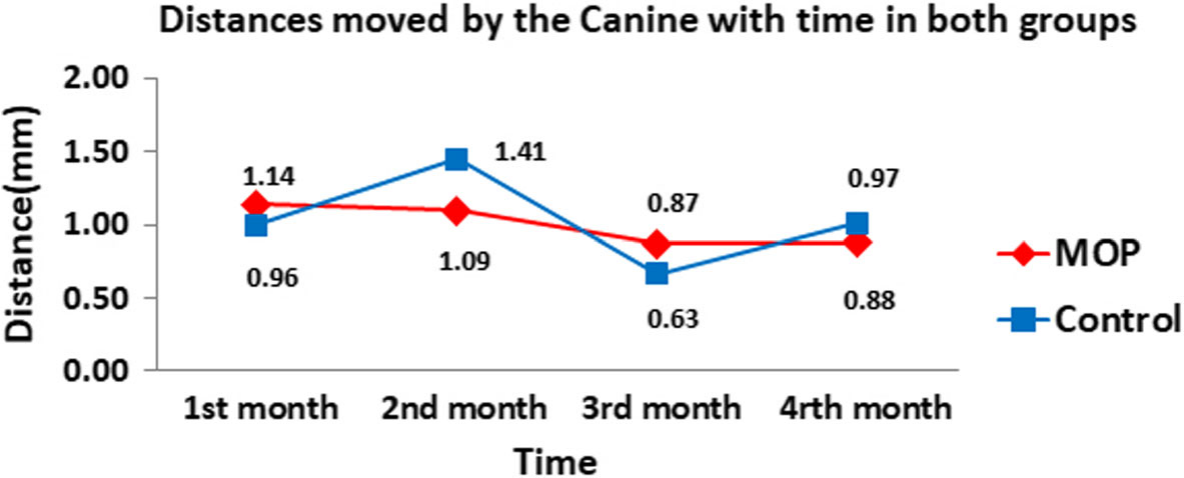
Time line chart representing the mean distances moved by the upper canines along the study time in the control and MOP sides

The mean distance moved by the canines in the control and MOP sides were 0.96, 1.41, 0.63, and 0.97 mm and 1.14, 1.09, 0.87, and 0.88 mm in the first, second, third, and fourth months respectively, with no significant difference (*P* > 0.05) at any observation time (Fig. 5). The mean rate of canine retraction was 0.99 ± 0.3 mm/month.

The mean total distance moved by the canines in the control and MOP sides were 3.97 mm and 3.98 mm respectively, with no significant difference (*P* > 0.05).

The mean anchorage loss of the upper first molars in the MOP and control sides were 0.49 ± 0.56 mm and 0.47 ± 0.42 mm respectively, with no significant difference (*P* > 0.05).

### Results of CBCT image assessment

All CBCT measurements showed excellent intra- and inter-observer reliability (CCC > 0.75).

#### Total distance moved by the canine (Table 1)

The distance moved by the canine cusp tip was greater in the MOP than the control side by 0.06 ± 0.72 mm, which was non-significant (*P* > 0.05). The distance moved by the canine center was greater in the MOP than the control side by 0.37 ± 0.63 mm, which was significant (*P* < 0.05). The distance moved by the canine apex was greater in the MOP than the control side by 0.47 ± 0.56 mm, which was a highly significant difference (*P* < 0.01).

**Table 1:** The total distances moved by the canine and the anchorage loss in the MOP and control sides (paired sample *t* test)

	CBCT measurement	Mean ± SD (mm)	Mean difference (MOP -CON)	SD	95% confidence interval of the difference		*P* value	
					Lower	Upper		
Total distances moved by the canine (mm)	Can-tip-FP (MOP)	3.34 ± 2.28	0.06	0.72	− 0.33	0.44	0.75764	*P* > 0.05 NS
	Can-tip-FP (CON)	3.29 ± 2.39						
	Can-C-FP (MOP)	2.13 ± 0.75	0.37	0.63	0.02	0.71	0.04121	*P < 0.05 S*
	Can-C-FP (CON)	1.76 ± 0.91						
	Can-apex-FP (MOP)	1.51 ± 0.95	0.47	0.56	0.17	0.77	0.00419	*P < 0.01 HS*
	Can-apex-FP (CON)	1.04 ± 0.87						
Anchorage loss (mm)	Mol tip-FP (MOP)	− 0.45 ± 0.59	− 0.18	0.43	− 0.41	0.05	0.11229	*P* > 0.05 NS
	Mol tip-FP (CON)	− 0.63 ± 0.49						
	Mol C-FP (MOP)	− 0.16 ± 0.55	− 0.09	1.84	− 1.07	0.89	0.84745	*P* > 0.05 NS
	Mol C-FP (CON)	− 0.25 ± 1.82						
	Mol apex-FP (MOP)	+ 0.13 ± 0.80	− 0.20	0.61	− 0.53	0.13	0.21226	*P* > 0.05 NS
	Mol apex-FP (CON)	− 0.07 ± 0.73						

*Can* upper canine, *MOP* micro-osteoperforation, *CON* control, *tip* cusp tip, *C* center, *apex* root apex, *Mol* upper first molar, *FP* frontal plane, *SD* standard deviation, *(− ve sign)* mesial molar movement, *(+ve sign)* distal molar movement, *SD* standard deviation, *NS* non-significant, *S* significant, *HS* highly significant

#### First molar anchorage loss (Table 1)

The mean difference of the anchorage loss by the mesiobuccal (MB) cusp tip, center, and root apex of the upper first molar was greater in the control than the MOP side by − 0.18 ± 0.43 mm, − 0.09 ± 1.84 mm, and − 0.20 ± 0.61 mm respectively, with no significant difference (*P* > 0.05).

#### Canine root resorption assessment (Table 2)

The difference between the pre- and post-retraction canine root resorption scores within the control and MOP groups was non-significant (*P* > 0.05).

**Table 2:** Canine root resorption scores (chi-squared test)

	Canine root resorption score	Pre-retraction canine root resorption		Post- retraction canine root resorption		Chi-squared	*P* value
		Frequency	Percent	Frequency	Percent		
Root resorption scores within the control side	Zero	28	82.4%	25	73.5%	1.17	0.76025 *P* > 0.05 NS
	One	2	5.9%	2	5.9%		
	Two	3	8.8%	6	17.6%		
	Three	1	2.9%	1	2.9%		
Root resorption scores within the MOP side	Zero	30	88.2%	23	67.6%	5.12	0.16290 *P* > 0.05 NS
	One	2	5.9%	3	8.8%		
	Two	2	5.9%	6	17.6%		
	Three	0	0.0%	2	5.9%		
Root resorption score difference between the MOP and control groups	Canine root resorption score difference (post-pre)	MOP group		Control group		Chi-squared	*P* value
	Zero	24	70.6%	30	88.2%	3.24	0.198 *P* > 0.05 NS
	One	5	14.7%	2	5.9%		
	Two	5	14.7%	2	5.9%		

NS non-significant

The difference between the pre- and post-retraction canine root resorption scores was calculated as follows: *zero*, the pre- and post- retraction scores were identical; *one*, the post-retraction score was 1 score greater than the pre-retraction score; *two*, the post-retraction score was 2 scores greater than the pre-retraction score. No significant difference was detected regarding canine root resorption score difference between the MOP and control sides (*P* > 0.05).

#### Pain assessment

The mean pain scores of the patients on the day of MOP procedure, after 24 h, 72 h, and 1 week were 4.88 ± 0.56, 2.69 ± 0.75, 1.31 ± 0.57, and 0.63 ± 0.43 respectively. Results of question 1 were as follows: 69% of the patients answered that the MOP side was more painful than the control side. Results of question 2 were as follows: 25% of the patients answered that MOP was more painful, 44% of the patients answered that extraction was more painful, 6% of the patients answered that they were equally painful, and 25% of the patients did not know which procedure was more painful.

## Discussion

The current study dealt with one of the crucial drawbacks of orthodontic treatment, which is its lengthy duration. Prolonged orthodontic treatment could be very disappointing for our patients, particularly for older age categories [1]. Appreciating the above fact, many studies were conducted to evaluate the effectiveness of surgical and non-surgical adjunctive procedures aiming to accelerate OTM [4, 5, 13–15]. Although proven to be effective [4], patients are unwilling to undergo corticotomies to reduce orthodontic treatment duration [23]. This might explain why corticotomy is not widely adopted in the orthodontic clinics. In an attempt to achieve the acceleratory effects of corticotomy and patient satisfaction, the flapless micro-osteoperforation (MOP) procedure was recently introduced [11]. Hence, the aim of this trial was to evaluate the effect of MOPs on the rate of OTM in a canine retraction model.

Adult females were recruited to rule out the effect of age and gender on OTM [24]. Malocclusions requiring bilateral extraction of the upper first premolars with maximum anchorage were selected, to allow investigation of the long-term effect of the RAP following the MOP procedure, which usually lasts for 2–3 months on average [4]. Being a minimally invasive flapless procedure, it was postulated that if MOP was performed on the day of premolar extraction, its effect would be easily obliterated. Therefore, 3 months time interval between premolar extraction and canine retraction commencement was scheduled to permit the return of the normal bone architecture in the extraction site [17]. The use of a TAD to perform the MOPs allowed standardization of the width and depth of the perforations.

Yang et al. [18] have shown that the maximum stress encountered during canine retraction was focused on its cervix at the distolabial side and added that distal corticotomy had similar biomechanical effects as a continuous circumscribing cut around the canine root. Based on their assumptions, the MOPs were only performed distal to the canine and vertically distributed along the cervical two thirds of the canine root length.

The incremental rate of canine retraction was measured on digital models using the 3 shape program, with proven reliability and validity for linear measurements [25]. Pre- and post-retraction CBCT images were used to detect the clinically invisible root movement. The specifications of the CBCT images used are considered one of the lowest radiation doses available, since a small field of view and brief scan time was chosen as prescribed by Feragalli et al. [26]. Furthermore, the patients included in the sample were not asked to do progress or post-treatment X-rays (which are routinely done) to reduce the radiation dose.

Upon comparing the mean canine retraction rate in the MOP and control sides, it was found to be almost identical (0.99 ± 0.3 mm/month), supporting the findings of Alkebsi et al. [12]. However, this was in disagreement with the results of Alikhani et al. [11] and Sivarajan et al. [27] who advocated that MOPs increased the rate of canine retraction compared to the control group. It is worth mentioning that the latter studies evaluated the canine retraction rate by direct measurements performed on the models or intraorally, which are less accurate than the indirect measurements performed on digital models.

Nevertheless, the difference in canine retraction rate fluctuation with time in both sides was interesting in the current study, where the MOP side showed more steady rate of OTM than the control side along the observation period. Movement of the canine in the control side typically showed alternating series of increased and decreased retraction rates corresponding to tipping and uprighting of the canine as explained by Andrews [28]. Yet, this pattern of canine retraction seemed to be less noticeable in the MOP side. By analyzing the canine root movement as measured from the CBCT images in both sides, it has been shown that the total distance moved by the center and apex of the canine was significantly greater in the MOP than the control side. Very much earlier, Reitan [29] emphasized that during canine retraction, the tooth acted as a two-armed lever, with the applied force concentrated at the alveolar crest, where the areas of hyalinization are concentrated. MOPs performed distal to the cervical two thirds of the canine root have probably decreased the resistance offered by the alveolar crest allowing greater root movement. However, the canine cusp tips moved a greater distance than the apices in both sides, indicating that canine retraction was mostly due to controlled tipping movement.

Very mild anchorage loss (< 1 mm) occurred in both the control and MOP sides, with no statistically significant difference. Minor anchorage loss, even with the utilization of absolute anchorage means, was documented formerly by El-Beialy et al. [30]

Results of the current study demonstrated that MOP neither increased nor decreased root resorption, in contrast to a recent systematic review which accused corticotomy of increasing the orthodontically induced root resorption [31].

The fundamental aim of all minimally invasive surgical procedures was to achieve accelerated OTM utilizing patient-friendly approaches; consequently, patients’ feedback was of utmost importance. The pain severity experienced by the patients ranged from mild to moderate pain that rapidly faded away after 1 week. Yet, the mean pain scores obtained in the current study were higher than those reported by Alikhani et al. [11].

## Limitations

The current study did not evaluate the effect of different numbers, sites, and repetition of MOP on the rate and type of tooth movement. The current study did not evaluate the effect of MOP on the overall treatment duration.

## Conclusions

Within the limitations of this study, the following conclusions could be withdrawn:

- Micro-osteoperforations (MOP) were not able to accelerate the rate of canine retraction; however, it seemed to facilitate root movement.
- MOP did not augment posterior anchorage.
- MOP did not increase nor decrease orthodontically induced root resorption. No long-term adverse effects on the alveolar mucosa were detected following the MOP procedure.
- Mild to moderate transient pain was experienced following the MOP procedures that almost disappeared 1 week later.

## Abbreviations

CBCT: Cone beam computed tomography
FP: Frontal plane
MB: Mesiobuccal
MOP: Micro-osteoperforation
OTM: Orthodontic tooth movement
TADs: Temporary anchorage devices

## References

[1] Skidmore KJ, Brook KJ, Thomson WM, Harding WJ (2006). Factors influencing treatment time in orthodontic patients. Am J Orthod Dentofac Orthop.

[2] Apajalahti S, Peltola JS (2007). Apical root resorption after orthodontic treatment-a retrospective study. Eur J Orthod.

[3] Diamanti-Kipioti A, Gusberti FA, Lang NP (1987). Clinical and microbiological effects of fixed orthodontic appliances. J Clin Periodontol.

[4] Yi J., Xiao J., Li H., Li Y., Li X., Zhao Z. (2017). Effectiveness of adjunctive interventions for accelerating orthodontic tooth movement: a systematic review of systematic reviews. Journal of Oral Rehabilitation.

[5] Dibart S, Sebaoun JD, Surmenian J. Piezocision: a minimally invasive, periodontally accelerated orthodontic tooth movement procedure. Compend Contin Educ Dent. 2009;30(6):342–4, 346. 19715011

[6] Teixeira CC, Khoo E, Tran J, Chartres I, Liu Y, Thant LM, Khabensky I, Gart LP, Cisneros G, Alikhani M (2010). Cytokine expression and accelerated tooth movement. J Dent Res.

[7] Safavi SM, Heidarpour M, Izadi SS, Heidarpour M (2012). Effects of flapless bur decortications on movement velocity of dogs’ teeth. Dent Res J (Isfahan).

[8] Tsai CY, Yang TK, Hsieh HY, Yang LY (2016). Comparison of the effects of micro-osteoperforation and corticision on the rate of orthodontic tooth movement in rats. Angle Orthod.

[9] Cheung T, Park J, Lee D, Kim C, Olson J, Javadi S, Lawson G, McCabe J, Moon W, Ting K, Hong C (2016). Ability of mini-implant-facilitated micro-osteoperforations to accelerate tooth movement in rats. Am J Orthod Dentofac Orthop.

[10] Lee Ji-Won, Cha Jung-Yul, Park Ki-Ho, Kang Yoon-Goo, Kim Su-Jung (2018). Effect of flapless osteoperforation-assisted tooth movement on atrophic alveolar ridge: Histomorphometric and gene-enrichment analysis. The Angle Orthodontist.

[11] Alikhani M, Raptis M, Zoldan B, Sangsuwon C, Lee YB, Alyami B, Corpodian C, Barrera LM, Alansari S, Khoo E, Teixeira C (2013). Effect of micro-osteoperforations on the rate of tooth movement. Am J Orthod Dentofacial Orthop.

[12] Alkebsi A, Al-Maaitah E, Al-Shorman H, Abu Alhaija E (2018). Three-dimensional assessment of the effect of micro-osteoperforations on the rate of toothmovement during canine retraction in adults with Class II malocclusion: a randomized controlled clinical trial. Am J Orthod Dentofac Orthop.

[13] Qamruddin I, Alam MK, Khamis MF, Husein A (2015). Minimally invasive techniques to accelerate the orthodontic tooth movement: a systematic review of animal studies. Biomed Res Int.

[14] Alfawal AM, Hajeer MY, Ajaj MA, Hamadah O, Brad B (2016). Effectiveness of minimally invasive surgical procedures in the acceleration of tooth movement: a systematic review and meta-analysis. Prog Orthod.

[15] Hoffmann Stefan, Papadopoulos Nikolaos, Visel Dominik, Visel Theresa, Jost-Brinkmann Paul-Georg, Präger Thomas Michael (2017). Influence of piezotomy and osteoperforation of the alveolar process on the rate of orthodontic tooth movement: a systematic review. Journal of Orofacial Orthopedics / Fortschritte der Kieferorthopädie.

[16] Aksakalli S, Calik B, Kara B, Ezirganli S (2015). Accelerated tooth movement piezocision and its periodontal-transversal effects in patients with class II malocclusion. Angle Orthod.

[17] Amler MH, Johnson PL, Salman I (1960). Histological and histochemical investigation of human alveolar socket healing in undisturbed extraction wounds. J Am Dent Assoc.

[18] Yang C, Wang C, Deng F, Fan Y (2015). Biomechanical effects of corticotomy approaches on dentoalveolar structures during canine retraction: a 3-dimensional finite element analysis. Am J Orthod Dentofac Orthop.

[19] McCaffery M, Beebe A (1989). Pain: clinical manual for nursing practice. The numeric pain rating scale. Pain.

[20] Lin HH, Chiang WC, Lo LJ, Wang CH (2013). A new method for the integration of digital dental models and cone-beam computed tomography images. Conf Proc IEEE Eng Med Biol Soc.

[21] Ziegler P, Ingervall B. A clinical study of maxillary canine retraction retraction spring and with sliding mechanics. Am J Orthod Dentofac Orthop. 1989;95(2):99–106. 10.1016/0889-5406(89)90388-0 2916474

[22] Malmgren O, Goldson L, Hill C, Orwin A, Petrini L, Lundberg M (1982). Root resorption after orthodontic treatment of traumatized teeth. Am J Orthod.

[23] Uribe F, Padala S, Allareddy V, Nanda R (2014). Patients’, parents’, and orthodontists’ perceptions of the need for and costs of additional procedures to reduce treatment time. Am J Orthod Dentofac Orthop.

[24] Dudic A, Giannopoulou C, Kiliaridis S (2013). Factors related to the rate of orthodontically induced tooth movement. Am J Orthod Dentofac Orthop.

[25] Luu NS, Nikolcheva LG, Retrouvey JM, Flores-Mir C, El-Bialy T, Carey JP, Major PW (2012). Linear measurements using virtual study models. Angle Orthod.

[26] Feragalli B, Rampado O, Abate C, Macrì M, Festa F, Stromei F, Caputi S, Guglielmi G (2017). Cone beam computed tomography for dental and maxillofacial imaging: technique improvement and low-dose protocols. Radiol Med.

[27] Sivarajan S, Doss JG, Papageorgiou SN, Cobourne MT, Wey MC. Mini-implant supported canine retraction with micro-osteoperforation: A split-mouth exploratory randomized clinical trial. Angle Orthod. 2019;89(2):183–9. 10.2319/011518-47.1 PMC8120871 30372126

[28] Andrews LF (1989). Straight wire: the concept and appliance.

[29] Reitan K (1963). Influence of variation in bone types and character on tooth movement. Eur Orthod Soc Tr.

[30] El-Beialy AR, Abou-El-Ezz AM, Attia KH, El-Bialy AM, Mostafa YA (2009). Loss of anchorage of miniscrews: a 3-dimensional assessment. Am J Orthod Dentofac Orthop.

[31] Haugland L, Kristensen KD, Lie SA, Vandevska-Radunovic V (2018). The effect of biologic factors and adjunctive therapies on orthodontically induced inflammatory root resorption: a systematic review and meta-analysis. Eur J Orthod.

